# Time-controlled adaptive ventilation in patients with ARDS—lack of protocol adherence: a systematic review

**DOI:** 10.1186/s13054-023-04340-w

**Published:** 2023-02-10

**Authors:** Stephan Katzenschlager, Christoph M. Simon, Patrick Rehn, Maurizio Grilli, Mascha O. Fiedler, Michael Müller, Markus A. Weigand, Benjamin Neetz

**Affiliations:** 1 Department of Anesthesiology, Heidelberg University Hospital, Im Neuenheimer Feld 420, 69120 Heidelberg, Germany; 2 University Center for ARDS and Weaning, Heidelberg University Hospital, Heidelberg, Germany; 3 Library, University Medical Center Mannheim, Mannheim, Germany; 4 Department of Pneumology and Critical Care Medicine, Thoraxklinik, Translational Lung Research Center Heidelberg (TLRC-H), Member of the German Center for Lung Research (DZL), University of Heidelberg, Heidelberg, Germany

To the editor,

Acute respiratory distress syndrome (ARDS) describes a polyetiological clinical picture characterized by diffuse alveolar damage and acute respiratory failure which has a prevalence of 10% in intensive care units [1]. One factor that influences mortality is the ventilatory strategy in invasively ventilated ARDS patients. Since the ARMA trial, there has been no multicenter randomized controlled trial that has been able to assign further mortality benefit to a particular ventilatory strategy [2]. The main goals of invasive ventilation strategies are to ensure an acceptable gas exchange while preventing ventilator-induced lung injury (VILI) therefore buying time for the lung to heal [3]. VILI is mainly attributed to repetitive opening and closing of lung units (atelectrauma) and/or cyclic overdistension of the lung (volutrauma) [4]. Different ventilation strategies in patients with acute respiratory distress syndrome (ARDS) have evolved over the course of the last decades. Currently, lung protective ventilation is defined as using low tidal volume (TV) (6 ml/kg predicted body weight) and a plateau pressure lower than 30 cmH_2_O to reduce VILI [5]. This approach significantly reduced mortality compared to a strategy with high TV and higher plateau pressures [3].

Time-controlled adaptive ventilation (TCAV) is a fairly novel protocol using the airway pressure release ventilation (APRV) mode. TCAV can be understood as continuous positive airway pressure (CPAP) which is briefly interrupted by a release phase, where gas is expelled. The CPAP-Phase, which accounts for approximately 90% of the total respiratory cycle, allows for time-dependent recruitment of the lungs. The release phase is timed to end when 75% of peak expiratory flow is reached. This approach has been shown to regain lung volume while preventing VILI in experimental studies [6].

The effect of APRV in comparison with other invasive mechanical ventilation strategies has been evaluated by several systematic reviews with meta-analysis [7–10] and found an association with reduced mortality and a lower length of intensive care unit (ICU) stay. But usage of the APRV mode does not imply that TCAV protocol is followed.

In order to assess the effect of TCAV on (1) mortality, (2) ventilator free days, (3) ICU length of stay, and (4) complications in comparison to other invasive mechanical ventilation strategies, we performed a systematic review with preplanned meta-analysis.

We developed a systematic review protocol following standard guidelines, registered the review on PROSPERO (CRD42022345754), and followed the PRISMA guideline.

A professional librarian conducted a systematic search since inception until 09. February 2022 using a combination of Mesh terms for “Respiratory Distress Syndrome” and “Continuous Positive Airway Pressure” alongside with terms for ARDS, APRV, TCAV, and lung protective ventilation, with the complete search term available in the Additional file 1. We limited our search only to patients with ARDS, without restrictions to etiology, age, gender or sociocultural circumstances and excluded animal studies. In order to present the latest data available, the search was conducted within peer reviewed databases and preprint servers. We considered prospective and retrospective clinical trials of patients with ARDS without restriction to the study type as eligible. The full list of screening criteria is available in the Additional file 1.

Our search yielded in 3459 publications. Screening was done by two authors in parallel following predefined inclusion and exclusion criteria, with differences solved by consensus with a third author.

After title and abstract screening, 111 articles were eligible for full text screening. No article fulfilled the predefined inclusion criteria for this systematic review. Main reasons for exclusion of studies were “no strict TCAV-protocol adherence”, while “no TCAV protocol defined” accounted for 39 articles. Most common deviation from the protocol was termination of expiratory flow not 75%. The full study flow diagram with reasons for exclusion is presented in Fig. 1. TCAV-protocol criteria are presented in the Additional file 1.

**Fig. 1:**
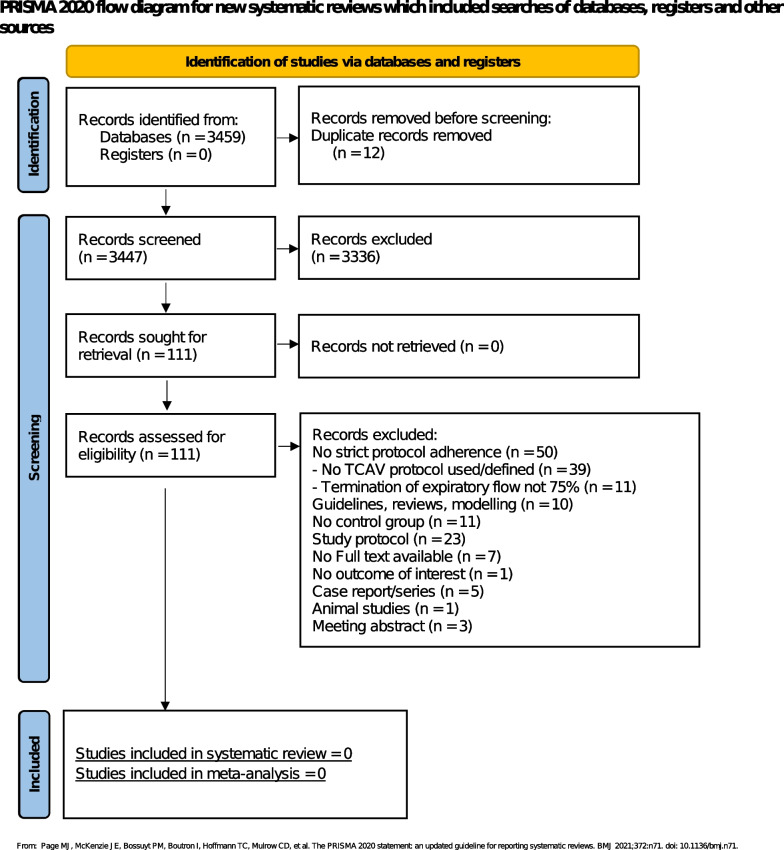
PRISMA flow diagram. *TCAV* time controlled adaptive ventilation

Despite the fact that no study was identified, that met the requirements of the TCAV protocol, there are important points to discuss. Several studies were close to the TCAV protocol but had to be excluded due to relevant details. Therefore, it seems reasonable to assume, that the TCAV protocol has been modified to meet current recommendations for lung-protective ventilation.

Hirshberg et al. tested two APRV-protocols which were deemed as not feasible because it was not possible to consistently ensure low TV [11]. Using the TCAV protocol, TV is generated in the release-phase and is dependable from the peak expiratory flow and the concomitant rate of flow decay, which is mainly determined by the elastance of the respiratory system (*E*_RS_). Since *E*_RS_ decreases when lung recruitment occurs, the higher TV within the TCAV-protocol will only occur, when lung recruits and *E*_RS_ is lowered. Due to this fact, higher TV are, more an indicator of response to therapy rather than a signal for potential harm. The rationale for this can be found in recent findings, which revealed that high tidal volumes are only harmful when *E*_RS_ is high [12]. Furthermore, Ibarra-Estrada et al. prolongated the release phase up until 50% of peak expiratory flow to decrease p_a_CO_2_ [13]. This can lead to a loss of alveolar stability. P_a_CO_2_ elimination should rather be controlled by modifying the length of the CPAP-phase.

Another modification of the protocol can be found in Ganesan et al. [14], where the pressure level of the CPAP phase was reduced to avoid increases in tidal volumes. This may result in loss of alveolar stability and could prevent lung opening [15]. The pressure level of the CPAP phase should rather be left until the inspiratory oxygen fraction falls below 0.4 to ensure a permanent stabilization of the end-inspiratory lung volume between functional residual capacity and total lung capacity.

In conclusion, strict adherence to the TCAV protocol in clinical trials has not yet been achieved due to clinical concerns in the respective studies. This systematic review presents absence of evidence on the effect of TCAV in comparison with other invasive mechanical strategies for patients with ARDS. Furthermore, it helps to overcome caveats concerning this novel protocol and emphasizes future clinical trials to test the TCAV protocol as it is safe to apply.

## Supplementary Information

**Additional file 1.** Additional information on screening criteria, TCAV protocol criteria, reasons for exclusions, and search term.

## Data Availability

The Endnote library is available from the corresponding author upon reasonable request. **Ethics approval and consent to participate** Not applicable.
