# Supplementary material for: Time-controlled adaptive ventilation in patients with ARDS—lack of protocol adherence: a systematic review
Source: Crit Care. 2023 Feb 10;27:57. doi: 10.1186/s13054-023-04340-w (PMC9921688; doi:10.1186/s13054-023-04340-w)
Supplement: Supplementary file 1 — Additional file 1. Additional information on screening criteria, TCAV protocol criteria, reasons for exclusions, and search term. [file 13054_2023_4340_MOESM1_ESM.docx]

Supplementary Material

**Time controlled adaptive ventilation in patients with ARDS – lack of protocol adherence: a systematic review**

Stephan Katzenschlager^1,2^*, Christoph M. Simon^1,2^, Patrick Rehn^1,2^, Maurizio Grilli^3^, Mascha O. Fiedler^1,2,4^, Michael Müller^2,4^, Markus A. Weigand^1,2,4^, Benjamin Neetz^2,4^

1 Department of Anesthesiology, Heidelberg University Hospital, Heidelberg, Germany

2 University Center for ARDS and Weaning, Heidelberg University Hospital, Heidelberg, Germany

3 Library, University Medical Center Mannheim, Mannheim, Germany

4 Department of Pneumology and Critical Care Medicine, Thoraxklinik, University of Heidelberg, Translational Lung Research Center Heidelberg (TLRC-H),

Member of the German Center for Lung Research (DZL), Heidelberg, Germany

* Corresponding author

Dr. med. univ. Stephan Katzenschlager

Department of Anaesthesiology, Heidelberg University Hospital, Germany

Im Neuenheimer Feld 420, 69120 Heidelberg, Germany

E-mail: [stephan.katzenschlager@med.uni-heidelberg.de](mailto:maximilian.dietrich@med.uni-heidelberg.de)

Phone: +49 6221 56 39683

**Table of contents**

[1. Screening Criteria 3](#_Toc123822641)

[2. TCAV protocol criteria 4](#_Toc123822642)

[3. List of excluded papers with reasons 5](#_Toc123822643)

[4. Search Term 13](#_Toc123822644)

# Screening Criteria

**Titel / Abstract**

Inclusion if suggestive of

- clinical study on ARDS patients

- Human patients

Exclusion if

- systematic review / meta-analysis

- animal study

- modeling

- laboratory study

**Full Text**

Inclusion if (all):

- TCAV was used (see also 2. TCAV protocol critera)

- Control group with mechanical ventilation

- At least one Outcome available: Ventilation free days at day 28/30, Mortality, ICU LOS, paO2/FiO2 at day 3, MAP at day 3, Complications (Pneumothorax, …)

Exclusion if (one):

- TCAV/APRV was not used/specifically defined

- No control group

- Outcome not of relevance

- No parameters to extract

- Review, Guideline, Case Report

- Animal study

# TCAV protocol criteria

- Ventilator mode: airway pressure release ventilation

- P_high_ set based on plateau pressure in previous mode and adjusted to stabilize lung volume

- P_low_ always zero cmH_2_O

- T_low_ as long as expiratory flow needs to reach 75% of its peak value

- T_high_ typically comprises around 90% of the entire respiratory cycle. Modifications should be made to alter respiratory rate with respect to control paCO_2_.

1. APRV Network - Setting APRV using the TCAV Method as a Rescue Strategy; downloaded at [www.tcavnetwork.org](http://www.tcavnetwork.org) at June 22, 2021
2. APRV Network - Standard Settings for APRV using the TCAV Method; downloaded at [www.tcavnetwork.org](http://www.tcavnetwork.org) at June 22, 2021
3. Nieman GF, Gatto LA, Andrews P, et al. Prevention and treatment of acute lung injury with time-controlled adaptive ventilation: physiologically informed modification of airway pressure release ventilation. Ann Intensive Care. 2020;10(1):3.; 10.1186/s13613-019-0619-3

4. Nieman GF, Andrews P, Satalin J, et al. Acute lung injury: how to stabilize a broken lung. Crit Care. 2018;22(1):136.; 10.1186/s13054-018-2051-8.

# List of excluded papers with reasons

| Article | Reason for exclusion |
| --- | --- |
| (2015). "More Patients Are Surviving ARDS." AARC Times 39(2): 70-70. | No full text available |
| (2021). Standard vs Alternative Vent Modes: What's the Difference? Alisa Veijo, California, American Association of Critical-Care Nurses. 41: 80-80. | Review |
| Abo-Hagar, H. H., et al. (2012). "Study of cardiac and hemodynamic changes with airway pressure release ventilation and pressure control ventilation in children with acute respiratory distress syndrome." European respiratory journal 40. | Review |
| Ali, N., et al. (2015). "Management Of Severe Ards With Extracorpeal Membrane Oxygenation And Airway Pressure Release Ventilation (aprv) - A Case Series." American journal of respiratory and critical care medicine 191. | Case series |
| Allan, P. F., et al. (2010). "Combat-associated drowning." J Trauma 69 Suppl 1: S179-187. | Case series |
| Allen, R., et al. (2020). "Interhospital Transfer of Critically Ill Patients Because of Coronavirus Disease 19–Related Respiratory Failure." Air Medical Journal 39(6): 498-501. | No strict protocol adherence:  No TCAV protocol used/defined |
| Amsterdam, A. M. C.-U. v., et al. (2017). Practice of Ventilation in Middle-Income Countries, https://ClinicalTrials.gov/show/NCT03188770. | Study protocol |
| Bauman, Z., et al. (2013). "Use of airway pressure release ventilation in acute respiratory distress syndrome." Critical care medicine. 41(12 SUPPL. 1): A85‐A86. | No strict protocol adherence:  No TCAV protocol used/defined |
| Beitler, J. R., et al. (2013). "Hypercapnia without acidemia, compared to hypercapnia with acidemia, is associated with reduced mortality in ARDS: results from the NHLBI ARDS network fluid and catheter treatment (FACT) trial." American journal of respiratory and critical care medicine 187. | No strict protocol adherence:  No TCAV protocol used/defined |
| Cane, R. D., et al. (1991). "Airway pressure release ventilation in severe acute respiratory failure." Chest 100(2): 460-463. | No full text available |
| ChiCtr (2021). "The effect of APRV and LTV on respiratory function in patients with different types of ARDS: a single-center, randomized controlled study." https://trialsearch.who.int/Trial2.aspx?TrialID=ChiCTR2100043657. | Study protocol |
| Chung, K., et al. (2009). "Best (burn center evaluation of standard therapies) ventilator mode study: a randomized controlled trial." Critical care medicine 37(12): A6‐. | Review |
| Ctri (2014). "Trial of an alternate mode of providing artificial breaths to children with very severe pneumonia." https://trialsearch.who.int/Trial2.aspx?TrialID=CTRI/2014/06/004677. | Study protocol |
| Ctri (2020). "Comparison two types of artificial respiration in severe COVID-19 patients." https://trialsearch.who.int/Trial2.aspx?TrialID=CTRI/2020/08/027502. | Study protocol |
| Ctri (2021). "To compare the effects of airway pressure release ventilation and pressure control ventilation modes of ventilation on patients admitted in ICU requiring ventilator support." https://trialsearch.who.int/Trial2.aspx?TrialID=CTRI/2021/05/033420. | Study protocol |
| Dart, B. W. t., et al. (2005). "Preliminary experience with airway pressure release ventilation in a trauma/surgical intensive care unit." J Trauma 59(1): 71-76. | No strict protocol adherence:  Termination of expiratory flow not 75% (was 40-50%) |
| Davis, K., Jr., et al. (1993). "Airway pressure release ventilation." Arch Surg 128(12): 1348-1352. | No strict protocol adherence:  No TCAV protocol used/defined |
| Denmark, U. o. S., et al. (2021). Ventilator Strategies in ICU Patients With COVID-19 - a National-wide Retrospective Observational Study, https://ClinicalTrials.gov/show/NCT05102630. | Study protocol |
| Diaz, Y., et al. (2011). "Early application of airway pressure release ventilation can benefit patients with Ali/ards." American journal of respiratory and critical care medicine 183(1 MeetingAbstracts). | No strict protocol adherence:  No TCAV protocol used/defined |
| Dolinay, T., et al. (2011). "Patients With Acute Lung Injury Benefit From Airway Pressure Release Ventilation." American journal of respiratory and critical care medicine 183(Meeting Abstracts): A1652. | No strict protocol adherence:  No TCAV protocol used/defined |
| Dong, M., et al. (2019). "Airway Pressure Release Ventilation: Is It Really Different in Adults and Children?" Am J Respir Crit Care Med 200(6): 788-789. | Review |
| Drake, T., et al. (2020). Outcome of hospitalisation for COVID-19 in patients with Interstitial Lung Disease: An international multicentre study, medRxiv. | No strict protocol adherence:  No TCAV protocol used/defined |
| Edgerton, C. A., et al. (2019). "Airway pressure release ventilation does not increase intracranial pressure in patients with traumatic brain injury with poor lung compliance." J Crit Care 50: 118-121. | No strict protocol adherence:  No TCAV protocol used/defined |
| Elie-Turenne, M., et al. (2010). "Early Application of a Novel Ventilator Strategy: APRV In Acute Respiratory Distress Syndrome." Annals of emergency medicine 56(3): S26-S26. | Review |
| Falkenhain, S. K., et al. (1992). "Improvement in cardiac output during airway pressure release ventilation." Crit Care Med 20(9): 1358-1360. | Case report |
| Fan, E., et al. (2008). "Review of a large clinical series: sedation and analgesia usage with airway pressure release and assist-control ventilation for acute lung injury." J Intensive Care Med 23(6): 376-383. | No strict protocol adherence:  No TCAV protocol used/defined |
| Fan, E. and T. E. Stewart (2006). "New modalities of mechanical ventilation: high-frequency oscillatory ventilation and airway pressure release ventilation." Clin Chest Med 27(4): 615-625; abstract viii-ix. | Review |
| Fletcher, J. J., et al. (2018). "Changes in Therapeutic Intensity Level Following Airway Pressure Release Ventilation in Severe Traumatic Brain Injury." J Intensive Care Med 33(3): 196-202. | No strict protocol adherence:  No TCAV protocol used/defined |
| Folk, J. J., et al. (2015). "Airway pressure release ventilation and respiratory failure during pregnancy. A report of three cases." J Reprod Med 60(1-2): 65-70. | Case series |
| Garner, W., et al. (1988). "Airway pressure release ventilation (APRV). A human trial." Chest 94(4): 779-781. | No strict protocol adherence:  No TCAV protocol used/defined |
| Gattinoni, L., et al. (1985). "Constant mean airway pressure with different patterns of positive pressure breathing during the adult respiratory distress syndrome." Bull Eur Physiopathol Respir 21(3): 275-279. | No strict protocol adherence:  No TCAV protocol used/defined |
| Gattinoni, L., et al. (1984). "The role of total static lung compliance in the management of severe ARDS unresponsive to conventional treatment." Intensive Care Med 10(3): 121-126. | No strict protocol adherence:  No TCAV protocol used/defined |
| Getafe, H. U. (2010). Third International Study on Mechanical Ventilation, https://ClinicalTrials.gov/show/NCT01093482. | No strict protocol adherence:  No TCAV protocol used/defined |
| González, M., et al. (2010). "Airway pressure release ventilation versus assist-control ventilation: a comparative propensity score and international cohort study." Intensive Care Med 36(5): 817-827. | No strict protocol adherence:  No TCAV protocol used/defined |
| Hering, R., et al. (2002). "Effects of spontaneous breathing during airway pressure release ventilation on renal perfusion and function in patients with acute lung injury." Intensive Care Med 28(10): 1426-1433. | No control group |
| Hirani, A., et al. (2009). "Airway pressure-release ventilation in pregnant patients with acute respiratory distress syndrome: a novel strategy." Respir Care 54(10): 1405-1408. | No control group |
| Hirshberg, E. L., et al. (2018). "Randomized Feasibility Trial of a Low Tidal Volume-Airway Pressure Release Ventilation Protocol Compared With Traditional Airway Pressure Release Ventilation and Volume Control Ventilation Protocols." Crit Care Med 46(12): 1943-1952. | No strict protocol adherence:  Termination of expiratory flow not 75% (was 50-75%), Plow > 0 to reduce tidal volumes |
| Hospital, K. E. A. (2017). Physiological Dead Space Measured by Volumetric Capnography in BiPAP and APRV, https://ClinicalTrials.gov/show/NCT03218943. | Study protocol |
| Ibarra-Estrada, M., et al. (2021). "Use of Airway Pressure Release Ventilation in Patients With Acute Respiratory Failure Due to Coronavirus Disease 2019: Results of a Single-Center Randomized Controlled Trial." Crit Care Med. | No strict protocol adherence:  Termination of expiratory flow not 75% (was 50-75%) |
| Irct20200509047376N (2020). "The effect of APRV ventilation mode on respiratory failure." https://trialsearch.who.int/Trial2.aspx?TrialID=IRCT20200509047376N1. | Study protocol |
| Jabaudon, M., et al. (2018). "Distinct Biological Effects of Time-Controlled Adaptive Ventilation in Pulmonary and Extrapulmonary Acute Respiratory Distress Syndrome: "One Small Step for Rats, One Giant Leap for Humans?"." Crit Care Med 46(6): 1038-1040. | Review |
| Jaramillo, P. M., et al. (2021). High-altitude is associated with better short-term survival in critically ill COVID-19 patients admitted to the ICU, medRxiv. | No strict protocol adherence:  No TCAV protocol used/defined |
| Kahn, M. R., et al. (2021). "High Incidence of Barotrauma in Patients With Severe Coronavirus Disease 2019." J Intensive Care Med 36(6): 646-654. | No outcome of interest |
| Kallet, R. H. (2011). "Patient-ventilator interaction during acute lung injury, and the role of spontaneous breathing: part 2: airway pressure release ventilation." Respir Care 56(2): 190-203; discussion 203-196. | Review |
| Kamath, S. S., et al. (2010). "Effects of airway pressure release ventilation on blood pressure and urine output in children." Pediatr Pulmonol 45(1): 48-54. | No strict protocol adherence:  No TCAV protocol used/defined |
| Kaplan, L. J., et al. (2001). "Airway pressure release ventilation increases cardiac performance in patients with acute lung injury/adult respiratory distress syndrome." Crit Care 5(4): 221-226. | No strict protocol adherence:  No TCAV protocol used/defined |
| Kawaguchi, A., et al. (2015). "Hemodynamic changes in child acute respiratory distress syndrome with airway pressure release ventilation: a case series." Clin Respir J 9(4): 423-429. | No control group |
| Kawaguchi, A., et al. (2009). "AIRWAY PRESSURE RELEASE VENTILATION FOR ACUTE RESPIRATORY DISTRESS SYNDROME IN PEDIATRICS." Critical care medicine 37(12): A465-A465. | Review |
| Kollisch-Singule, M. (2016). "Mechanical Breath Profile of Airway Pressure Release Ventilation: The Effect on Alveolar Recruitment and Microstrain in Acute Lung Injury (vol 149, pg 1138, 2014)." Jama Surgery 151(12): 1193-1193. | Animal study |
| Kotani, T., et al. (2016). "Pressure-controlled inverse ratio ventilation as a rescue therapy for severe acute respiratory distress syndrome." Springerplus 5(1): 716. | No strict protocol adherence:  No TCAV protocol used/defined |
| Krishnan, J. and W. Morrison (2007). "Airway pressure release ventilation: a pediatric case series." Pediatr Pulmonol 42(1): 83-88. | No strict protocol adherence:  Termination of expiratory flow not 75% (was 50-75%) |
| Kupfer, Y., et al. (2010). "Use Of Airway Pressure Release Ventilation As A Rescue Ventilatory Mode After Failure Of Pressure Regulated Volume Control For Patients With Severe ARDS." American journal of respiratory and critical care medicine 181. | Review |
| Küçük, M. P., et al. (2022). "The effect of preemptive airway pressure release ventilation on patients with high risk for acute respiratory distress syndrome: a randomized controlled trial." Braz J Anesthesiol 72(1): 29-36. | No strict protocol adherence:  Termination of expiratory flow not 75% (Tlow adjusted according to the PCO_2_) |
| Kyle, W. E., et al. (2010). "APRV Vs. ARDSnet Protocol Ventilation For ARDS/ALI." American journal of respiratory and critical care medicine 181(Meeting Abstracts): A1691. | Meeting abstract |
| Labelle, T. O. M., et al. (2013). "CONTENTIOUS RECRUITMENT WITH AIRWAY PRESSURE RELEASE VENTILATION AND PRONE POSITION IN PATIENTS WITH SEVERE ACUTE RESPIRATORY DISTRESS SYNDROME." Intensive care medicine 39: S309-S309. | Meeting abstract |
| Lalgudi Ganesan, S., et al. (2018). "Airway Pressure Release Ventilation in Pediatric Acute Respiratory Distress Syndrome. A Randomized Controlled Trial." Am J Respir Crit Care Med 198(9): 1199-1207. | No strict protocol adherence:  P_high_ adjusted to reduce VTs |
| Lee, S. J., et al. (2020). "Airway Pressure Release Ventilation Combined With Prone Positioning in Acute Respiratory Distress Syndrome: Old Tricks New Synergy: A Case Series." A A Pract 14(8): e01231. | Case series |
| Li, J. Q., et al. (2016). "Clinical research about airway pressure release ventilation for moderate to severe acute respiratory distress syndrome." Eur Rev Med Pharmacol Sci 20(12): 2634-2641. | No strict protocol adherence:  No TCAV protocol used/defined |
| Lim, J., et al. (2016). "Characteristics and outcomes of patients treated with airway pressure release ventilation for acute respiratory distress syndrome: A retrospective observational study." J Crit Care 34: 154-159. | No control group |
| Liu, L., et al. (2009). "Practical use of airway pressure release ventilation for severe ARDS--a preliminary report in comparison with a conventional ventilatory support." Hiroshima J Med Sci 58(4): 83-88. | No strict protocol adherence:  No TCAV protocol used/defined |
| Loh, F. and Y. H. Mok (2009). "USE OF AIRWAY PRESSURE RELEASE VENTILATION IN CHILDREN WITH NOVEL INFLUENZAA H1N1 STRAIN ACUTE LUNG INJURY." Critical care medicine 37(12): A188-A188. | Meeting abstract |
| Mahmoud, O., et al. (2021). "Utilization of Airway Pressure Release Ventilation as a Rescue Strategy in COVID-19 Patients: A Retrospective Analysis." J Intensive Care Med 36(10): 1194-1200. | No strict protocol adherence:  No TCAV protocol used/defined |
| Mancebo, J., et al. (1993). "Effects of airway pressure release ventilation on respiratory workload and gas exchange in patients with acute respiratory failure." Medicina intensiva. 17(7): 426‐434. | No full text available |
| Manjunath, V., et al. (2021). "Is airway pressure release ventilation, a better primary mode of post-operative ventilation for adult patients undergoing open heart surgery? A prospective randomised study." Ann Card Anaesth 24(3): 288-293. | No strict protocol adherence:  No TCAV protocol used/defined |
| Maung, A. A., et al. (2012). "Compared to conventional ventilation, airway pressure release ventilation may increase ventilator days in trauma patients." J Trauma Acute Care Surg 73(2): 507-510. | No strict protocol adherence:  No TCAV protocol used/defined |
| Maxwell, R. A., et al. (2010). "A randomized prospective trial of airway pressure release ventilation and low tidal volume ventilation in adult trauma patients with acute respiratory failure." J Trauma 69(3): 501-510; discussion 511. | No strict protocol adherence:  Termination of expiratory flow not 75% (was 25-75%) |
| Nct (2008). "Airway Pressure Release Ventilation in Acute Lung Injury." https://clinicaltrials.gov/show/NCT00750204. | Study protocol |
| Nct (2008). "Airway Pressure Release Ventilation (APRV) Compared to ARDSnet Ventilation." https://clinicaltrials.gov/show/NCT00793013. | Study protocol |
| Nct (2009). "Biomarkers of Lung Injury With Low Tidal Volume Ventilation Compared With Airway Pressure Release Ventilation." https://clinicaltrials.gov/show/NCT01038531. | Study protocol |
| Nct (2010). "Airway Pressure Release Ventilation (APRV) Versus AC/VC Conventional Ventilation." https://clinicaltrials.gov/show/NCT01339533. | Study protocol |
| Nct (2011). "Extracorporeal Membrane Oxygenation for Severe Acute Respiratory Distress Syndrome." https://clinicaltrials.gov/show/NCT01470703. | No strict protocol adherence:  No TCAV protocol used/defined |
| Nct (2013). "Acute Lung Injury Ventilator Evaluation (ALIVE)." https://clinicaltrials.gov/show/NCT01901354. | Study protocol |
| Nct (2013). "Early Spontaneous Breathing in Acute Respiratory Distress Syndrome." https://clinicaltrials.gov/show/NCT01862016. | Study protocol |
| Nct (2018). "A Multiple Centre,Random Control Study : early Use of Airway Pressure Release Ventilation (APRV) Plus Protocol in ARDS." https://clinicaltrials.gov/show/NCT03549910. | Study protocol |
| Nct (2019). "Effect of Two Modes of Mechanical Ventilation on Metabolic Demands and Respiratory Mechanics." https://clinicaltrials.gov/show/NCT04205422. | Study protocol |
| Nct (2019). "Airway Pressure Release Ventilation for Moderate-to-severe Acute Respiratory Distress Syndrome." https://clinicaltrials.gov/show/NCT04156438. | Study protocol |
| Nct (2019). "Comparison of Volume Assist Control, Dual Mode and Airway Pressure Release Ventilation." https://clinicaltrials.gov/show/NCT04196738. | Study protocol |
| Nct (2020). "Early PReserved SPONtaneous Breathing Activity in Mechanically Ventilated Patients With ARDS (PReSPON)." https://clinicaltrials.gov/show/NCT04228471. | Study protocol |
| Nct (2020). "Early Use of Airway Pressure Release Ventilation (APRV) in ARDS." https://clinicaltrials.gov/show/NCT04221737. | Study protocol |
| Nct (2021). "Ventilatory Strategy Based on Ultrasound Lung Morphology in Patients With Focal ARDS." https://clinicaltrials.gov/show/NCT04966624. | Study protocol |
| Neumann, P., et al. (2002). "Influence of different release times on spontaneous breathing pattern during airway pressure release ventilation." Intensive Care Med 28(12): 1742-1749. | No strict protocol adherence:  No TCAV protocol used/defined |
| Ning, B., et al. (2020). "The effect of high-frequency oscillatory ventilation or airway pressure release ventilation on children with acute respiratory distress syndrome as a rescue therapy." Transl Pediatr 9(3): 213-220. | No strict protocol adherence:  Termination of expiratory flow not 75% (was 50-75%) |
| Pandya, K., et al. (2013). "Improved Oxygenation and Lung Recruitment in Infants With Ali/Ards Using Aprv." Critical care medicine 41(12). | No control group |
| Pequignot, B., et al. (2020). "Evaluation of a 6-hour trial of inverse-ratio Airway Pressure Release Ventilation (APRV) in Covid-19 pneumonia." Intensive care medicine experimental 8(SUPPL 2). | No control group |
| Putensen, C., et al. (1999). "Spontaneous breathing during ventilatory support improves ventilation-perfusion distributions in patients with acute respiratory distress syndrome." Am J Respir Crit Care Med 159(4 Pt 1): 1241-1248. | No strict protocol adherence:  No TCAV protocol used/defined |
| Putensen, C., et al. (2001). "Long-term effects of spontaneous breathing during ventilatory support in patients with acute lung injury." Am J Respir Crit Care Med 164(1): 43-49. | No strict protocol adherence:  No TCAV protocol used/defined |
| Räsänen, J., et al. (1991). "Airway pressure release ventilation during acute lung injury: a prospective multicenter trial." Crit Care Med 19(10): 1234-1241. | No strict protocol adherence:  No TCAV protocol used/defined |
| Richard, J. C., et al. (2013). "Potentially harmful effects of inspiratory synchronization during pressure preset ventilation." Intensive Care Med 39(11): 2003-2010. | No strict protocol adherence:  No TCAV protocol used/defined |
| Rittayamai, N., et al. (2017). "Effect of inspiratory synchronization during pressure-controlled ventilation on lung distension and inspiratory effort." Ann Intensive Care 7(1): 100. | No strict protocol adherence:  No TCAV protocol used/defined |
| Rouby, J. J., et al. (1992). "Continuous positive airway pressure (CPAP) vs. intermittent mandatory pressure release ventilation (IMPRV) in patients with acute respiratory failure." Intensive Care Med 18(2): 69-75. | No strict protocol adherence:  No TCAV protocol used/defined |
| Rozé, H., et al. (2017). "Spontaneous breathing (SB) using airway pressure-release ventilation (APRV) in patients under extracorporeal-membrane oxygenation (ECMO) for acute respiratory distress syndrome (ARDS)." Intensive Care Med 43(12): 1919-1920. | No control group |
| Sathyanarayanan, S. P., et al. (2021). "AIRWAY PRESSURE RELEASE VENTILATION USE IN COVID-19 ARDS: A SINGLE CENTER OBSERVATIONAL STUDY." Chest 160(4): 1089A-1090A. | No strict protocol adherence:  No TCAV protocol used/defined |
| Shiber, J., et al. (2009). "APRV IS ASSOCIATED WITH A LOW RATE OF ARDS IN HIGH-RISK TRAUMA PATIENTS." Critical care medicine 37(12): A185-A185. | No full text available |
| Song, S., et al. (2016). "[The clinical effect of airway pressure release ventilation for acute lung injury/acute respiratory distress syndrome]." Zhonghua Wei Zhong Bing Ji Jiu Yi Xue 28(1): 15-21. | No full text available |
| Sundar, K. M., et al. (2012). "Clinical course of ICU patients with severe pandemic 2009 influenza A (H1N1) pneumonia: single center experience with proning and pressure release ventilation." J Intensive Care Med 27(3): 184-190. | No strict protocol adherence:  No TCAV protocol used/defined |
| Sydow, M., et al. (1994). "Long-term effects of two different ventilatory modes on oxygenation in acute lung injury. Comparison of airway pressure release ventilation and volume-controlled inverse ratio ventilation." Am J Respir Crit Care Med 149(6): 1550-1556. | No control group |
| Taha, A. (2010). "TRANSITION FROM CMV TO APRV MAY FACILITATE THE WEANING PROCESS IN PATIENTS WITH SEVERE ARDS." Intensive care medicine 36: S348-S348. | No control group |
| Tctr (2017). "A randomized controlled trial of airway pressure release ventilation and low tidal volume ventilation in pediatric acute respiratory distress syndrome." https://trialsearch.who.int/Trial2.aspx?TrialID=TCTR20170320002. | Study protocol |
| Testerman, G. M., et al. (2013). "Airway pressure release ventilation in morbidly obese surgical patients with acute lung injury and acute respiratory distress syndrome." Am Surg 79(3): 242-246. | No control group |
| University, A. (2017). Mechanical Ventilation in Multiple Fracture Ribs, https://ClinicalTrials.gov/show/NCT03314701. | Study protocol |
| Varpula, T., et al. (2003). "Combined effects of prone positioning and airway pressure release ventilation on gas exchange in patients with acute lung injury." Acta Anaesthesiol Scand 47(5): 516-524. | No strict protocol adherence:  No TCAV protocol used/defined |
| Varpula, T., et al. (2009). "The effects of ventilatory mode on lung aeration assessed with computer tomography: a randomized controlled study." J Intensive Care Med 24(2): 122-130. | No strict protocol adherence:  No TCAV protocol used/defined |
| Varpula, T., et al. (2004). "Airway pressure release ventilation as a primary ventilatory mode in acute respiratory distress syndrome." Acta Anaesthesiol Scand 48(6): 722-731. | No strict protocol adherence:  No TCAV protocol used/defined |
| Wrigge, H., et al. (2001). "Cardiorespiratory effects of automatic tube compensation during airway pressure release ventilation in patients with acute lung injury." Anesthesiology 95(2): 382-389. | No strict protocol adherence:  No TCAV protocol used/defined |
| Xu, L., et al. (2010). "[The lung protection strategy under the support of extracorporeal membrane oxygenation in patients suffering from influenza A H1N1]." Zhongguo Wei Zhong Bing Ji Jiu Yi Xue 22(3): 150-152. | No full text available |
| Yehya, N., et al. (2014). "High frequency oscillation and airway pressure release ventilation in pediatric respiratory failure." Pediatr Pulmonol 49(7): 707-715. | No strict protocol adherence:  Termination of expiratory flow not 75% (was 50-75%) |
| Yener, N. and M. Üdürgücü (2020). "Airway Pressure Release Ventilation as a Rescue Therapy in Pediatric Acute Respiratory Distress Syndrome." Indian J Pediatr 87(11): 905-909. | No control group |
| Yoshida, T., et al. (2009). "The impact of spontaneous ventilation on distribution of lung aeration in patients with acute respiratory distress syndrome: airway pressure release ventilation versus pressure support ventilation." Anesth Analg 109(6): 1892-1900. | No strict protocol adherence:  Termination of expiratory flow not 75% (was 50-75%), P_high_ set to target a specific VT |
| Zhang, N. X., et al. (2005). "[Clinical comparative study of airway pressure release ventilation and continuous positive airway pressure ventilation]." Zhongguo Wei Zhong Bing Ji Jiu Yi Xue 17(8): 481-483. | No full text available |
| Zhou, Y., et al. (2017). "Early application of airway pressure release ventilation may reduce the duration of mechanical ventilation in acute respiratory distress syndrome." Intensive Care Med 43(11): 1648-1659. | No strict protocol adherence:  Termination of expiratory flow not 75% (was >50%), P_low_ = 50 cmH_2_O |
| Zorbas, J. S., et al. (2021). "Airway pressure release ventilation in mechanically ventilated patients with COVID-19: a multicenter observational study." Acute Crit Care 36(2): 143-150. | No strict protocol adherence:  No TCAV protocol used/defined |

# Search Term

PubMed

| **Records number** | **Date** |
| --- | --- |
| 2059 | 09.02.2022 |

1. P (72982)

| **"Respiratory Distress Syndrome"[Mesh] OR**  respiratory distress syndrom*[tw] OR  ARDS*[tw] OR  "Shock Lung"[tw] OR  acute respiratory fail*[tw] OR  acute lung fail*[tw] OR  acute lung injur*[tw] |  |
| --- | --- |

2. I (16944)

| **"Continuous Positive Airway Pressure"[Mesh] OR**  Continuous Positive Airway Pressure*[tw] OR  CPAP*[tw] OR  nCPAP*[tw] OR  "Airway Pressure Release Ventilation"[tw] OR  APRV*[tw] OR |  |
| --- | --- |
| "Time controlled adaptive ventilation"[tw] OR  TCAV*[tw] OR |  |
| "small tidal volume ventilation"[tw] OR  "low tidal volume ventilation"[tw] |  |

3. Humans (4955191)

| NOT ("Animals"[Mesh] NOT "humans"[mesh]) |  |
| --- | --- |

4. (2462)

1 AND 2

5. (2334) Humans

Cochrane Library

| **Records number** | **Date** |
| --- | --- |
| 1114 | 09.02.2022 |

1. P (8528)

| [mh "Respiratory Distress Syndrome"] OR  respiratory NEXT distress NEXT syndrom*:ti,ab,kw OR  ARDS*:ti,ab,kw OR  Shock NEXT Lung:ti,ab,kw OR  acute NEXT respiratory NEXT fail*:ti,ab,kw OR  acute NEXT lung NEXT fail*:ti,ab,kw OR  acute NEXT lung NEXT injur*:ti,ab,kw |  |
| --- | --- |

2. I (6515)

| [mh "Continuous Positive Airway Pressure"] OR  Continuous NEXT Positive NEXT Airway NEXT Pressure*:ti,ab,kw OR  CPAP*:ti,ab,kw OR  nCPAP*:ti,ab,kw OR  Airway NEXT Pressure NEXT Release NEXT Ventilation:ti,ab,kw OR  APRV*:ti,ab,kw OR  Time NEXT controlled NEXT adaptive NEXT ventilation:ti,ab,kw OR  TCAV*:ti,ab,kw OR  small NEXT tidal NEXT volume NEXT ventilation:ti,ab,kw OR  low NEXT tidal NEXT volume NEXT ventilation:ti,ab,kw |  |
| --- | --- |

Search strings

(as in the table above)

Web of Science Core Collection

| **Records number** | **Date** |
| --- | --- |
| 1196 | 09.02.2022 |

1. P (49509)

| "respiratory distress syndrom*" OR  "ARDS*" OR  "Shock Lung" OR  "acute respiratory fail*" OR  "acute lung fail*" OR  "acute lung injur*" |  |
| --- | --- |

2. I (14821)

| "Continuous Positive Airway Pressure*" OR  "CPAP*" OR  "nCPAP*" OR  "Airway Pressure Release Ventilation" OR  "APRV*" OR  "Time controlled adaptive ventilation" OR  "TCAV*" OR  "small tidal volume ventilation" OR  "low tidal volume ventilation" |  |
| --- | --- |

Search Strings

**P**

TI=("respiratory distress syndrom*" OR "ARDS*" OR "Shock Lung" OR "acute respiratory fail*" OR "acute lung fail*" OR "acute lung injur*") OR AB=("respiratory distress syndrom*" OR "ARDS*" OR "Shock Lung" OR "acute respiratory fail*" OR "acute lung fail*" OR "acute lung injur*")

**I**

TI=("Continuous Positive Airway Pressure*" OR "CPAP*" OR "nCPAP*" OR "Airway Pressure Release Ventilation" OR "APRV*" OR "Time controlled adaptive ventilation" OR "TCAV*" OR "small tidal volume ventilation" OR "low tidal volume ventilation") OR AB=("Continuous Positive Airway Pressure*" OR "CPAP*" OR "nCPAP*" OR "Airway Pressure Release Ventilation" OR "APRV*" OR "Time controlled adaptive ventilation" OR "TCAV*" OR "small tidal volume ventilation" OR "low tidal volume ventilation")

**3. (1196)**

1 AND 2

CINAHL

| **Records number** | **Date** |
| --- | --- |
| 658 | 09.02.2022 |

1. P (15197)

| TI ("respiratory distress syndrom*" OR  "ARDS*" OR  "Shock Lung" OR  "acute respiratory fail*" OR  "acute lung fail*" OR  "acute lung injur*") OR  AB ("respiratory distress syndrom*" OR  "ARDS*" OR  "Shock Lung" OR  "acute respiratory fail*" OR  "acute lung fail*" OR  "acute lung injur*") |  |
| --- | --- |

2. I (5495)

| TI ("Continuous Positive Airway Pressure*" OR  "CPAP*" OR  "nCPAP*" OR  "Airway Pressure Release Ventilation" OR  "APRV*" OR  "Time controlled adaptive ventilation" OR  "TCAV*" OR  "small tidal volume ventilation" OR  "low tidal volume ventilation") OR  AB ("Continuous Positive Airway Pressure*" OR  "CPAP*" OR  "nCPAP*" OR  "Airway Pressure Release Ventilation" OR  "APRV*" OR  "Time controlled adaptive ventilation" OR  "TCAV*" OR  "small tidal volume ventilation" OR  "low tidal volume ventilation") |  |
| --- | --- |

**3. (658)**

1 AND 2

Clinical Trial Gov

<http://www.clinicaltrials.gov/>

| **Records number** | **Date** |
| --- | --- |
| 326 | 09.02.2022 |

1. P (3207)

| "respiratory distress syndrom" OR  ARDS OR  "Shock Lung" OR  "acute respiratory failure" OR  "acute lung failure" OR  "acute lung injury" |  |
| --- | --- |

2. I (1807)

| "Continuous Positive Airway Pressure" OR  CPAP OR  nCPAP OR  "Airway Pressure Release Ventilation" OR  APRV OR  "Time controlled adaptive ventilation" OR  TCAV OR  "small tidal volume ventilation" OR  "low tidal volume ventilation" |  |
| --- | --- |

Search strings

|  |  | **Records number** |
| --- | --- | --- |
| **P** | ("respiratory distress syndrom" OR ARDS OR "Shock Lung" OR "acute respiratory failure" OR "acute lung failure" OR "acute lung injury")  AND |  |
| **I** | ("Continuous Positive Airway Pressure" OR CPAP OR nCPAP OR "Airway Pressure Release Ventilation" OR APRV OR "Time controlled adaptive ventilation" OR TCAV OR "small tidal volume ventilation" OR "low tidal volume ventilation") |  |

International Clinical Trials Registry Platform ICTRP (WHO Trials)

<https://trialsearch.who.int/Default.aspx>

| **Records number** | **Date** |
| --- | --- |
| 42 | 09.02.2022 |

1. P ()

| respiratory distress syndrom OR  ARDS OR  Shock Lung OR  acute respiratory failure OR  acute lung failure OR  acute lung injury |  |
| --- | --- |

2. I ()

| Continuous Positive Airway Pressure OR  CPAP OR  nCPAP OR  Airway Pressure Release Ventilation OR  APRV OR  Time controlled adaptive ventilation OR  TCAV OR  small tidal volume ventilation OR  low tidal volume ventilation |  |
| --- | --- |

Search strings

|  | **Search strings** | **Records number** |
| --- | --- | --- |
| **Condition** |  | 1145 |
| **Intervention** |  | 1297 |
